# Nucleotide variation in Foxp3 gene and prognosis of bladder cancer: a case-control study

**DOI:** 10.3389/fonc.2025.1506900

**Published:** 2025-01-28

**Authors:** Qin Li, Yan Zhang, Min Su, Yaping Song, Yanyun Wang, Bin Zhou, Lin Zhang

**Affiliations:** ^1^ Laboratory of Molecular Translational Medicine, Center for Translational Medicine, Key Laboratory of Birth Defects and Related Diseases of Women and Children (Sichuan University), Ministry of Education, West China Second University Hospital, Sichuan University, Chengdu, Sichuan, China; ^2^ Department of Pathology, West China Second University Hospital, Sichuan University, Chengdu, Sichuan, China

**Keywords:** Foxp3, polymorphism, prognosis, non-muscle-invasive bladder cancer, tumor microenvironment

## Abstract

**Background:**

Numerous researches have investigated the correlation between single nucleotide polymorphisms (SNPs) in the transcription factor forkhead box protein 3 (Foxp3) gene and the development of various cancers. However, the relationship of Foxp3 polymorphism and bladder cancer (BC) remain unclear.

**Method:**

This hospital-based case-control study enrolled a total of 316 patients diagnosed with BC and 643 healthy controls. Two Foxp3 SNPs (rs3761548 C/A, rs5902434 del/ATT) were selected, and genotyping of the samples was performed using the polymerase chain reaction-restriction fragment length polymorphism (PCR-RFLP) technique. SPSS and online SNPstats software were used to determine the disparities between groups.

**Results:**

For the rs3761548 C/A polymorphism, patients with the CA/AA genotype showed a notable decrease in the case group (22.1% versus 34.8%, P = 0.003, OR = 0.61, 95%CI = 0.44-0.85), and the heterozygous CA genotype presented a distinctly lower risk for BC (P = 0.0003, OR = 0.43, 95%CI = 0.26-0.70). Notably, individuals who were homozygous for the AA genotype demonstrated a markedly lower overall survival (OS) rate compared to those with the CC/CA genotypes (P = 0.03, OR = 5.89, 95%CI = 1.23-28.15), after adjusting for factors such as age, gender, smoking status, tumor grade, metastasis, and clinical stage. For the rs5902434 del/ATT polymorphism, a decreased risk was observed across the codominant and over-dominant models with statistical significance (codominant model: P = 0.01, OR = 0.61, 95%CI = 0.42-0.89; over-dominant model: P = 0.004, OR = 0.60, 95%CI = 0.42-0.85), and no significant association was observed between the rs5902434 polymorphism and patient’s OS rate.

**Conclusions:**

Our findings indicate that Foxp3 polymorphisms may be associated with BC susceptibility, and that rs3761548 could potentially serve as an independent risk factor for the OS rate.

## 1 Introduction

Bladder cancer (BC) ranks the tenth most commonly diagnosed cancer worldwide as reported by the International Agency for Research on Cancer (IARC), with 573,278 new cases as well as 212,536 deaths in 2020 (1). Notably, China has the highest incidence and mortality rates for BC in Asia among 2020, with 85694 new cases and 39393 deaths (2). Its epidemiological distributions varies among sex, area and age in China. The incidence and mortality rates of BC are disproportionately higher in men, approximately four times that of women, which in line with the global pattern (1). The urban patients are 1.4 times as high as that in rural areas, and the increased incidence through the country is partly owing to the aging after 45 years old and the abusing of cigarette in recent years (3). Consequently, evaluating the accurate and cost-effective screening biomarkers and controlling the tobacco use have become breakthroughs in solving the problem. Non-muscle-invasive bladder cancer (NMIBC) constitutes approximately 75% of all BCs and typically has a more favorable prognosis. In contrast, muscle-invasive bladder cancer (MIBC) presents a more lethal phenotype, with a 5-year survival rate of approximately 50%, even with aggressive treatment strategies (4, 5). Encouragingly, advancements in treatment modalities, including endoscopic resection, adjuvant chemotherapy instillation, and intravesical immunotherapy, have led to a significant reduction in BC mortality rates (6).

Tobacco smoking is recognized as one of the most significant carcinogens associated with BC, although Schistosoma haematobium infection and other risk factors may also play a substantial role in certain populations (7, 8). Recent genome-wide association studies have explored the interactions between smoking and single nucleotide polymorphisms (SNPs) in BC patients, yet no conclusive link has been established (9). Genetic factors are increasingly acknowledged as critical elements in the pathogenesis of BC. Numerous family-based studies have indicated that individuals with a family history of BC are approximately twice as likely to develop the disease, underscoring the familial aggregation of BC and its significant genetic component (10–13). Furthermore, the unchecked proliferation of tumor cells is closely associated with the tumor’s ability to evade immune surveillance, a process that involves the participation of regulatory immune cell populations (14, 15).

Regulatory T (Treg) cells are pivotal for the mechanisms of tumor immune based on their immunosuppressive functions. They may contribute to the failure of tumor immunotherapy (16, 17). Specifically, CD4+ Treg cells express the transcription factor gene known as forkhead box P3 (Foxp3), located on the X chromosome at Xp11.23, and regulates T cell activation and function via downregulating the cytokine production (18, 19). The polymorphic variants within the Foxp3 gene induce autoimmune diseases, potentially through decreasing the number of functional CD4+CD25+ Tregs (20). These genetic variations may also influence tumor progression by regulating the tumor microenvironment. Numerous researches have investigated the correlation between SNPs in the Foxp3 gene and the development of various cancers (21).

Tregs are known to regulate the proliferation and activation of immune cells in the tumor microenvironment, which can affect the patient survival rates. The decrease in Treg cell function leads to a disruption in the immune homeostasis (20). However, increasing evidence suggests that Tregs have paradoxical prognostic effects on BC, which has partly been attributed to the misidentification of specific biomarkers, and the inflammatory profile of the tumor (22). Immunohistochemical studies have demonstrated an increase in the number of ghrelin-induced Foxp3+ Treg cells within BC tissues, which suppress the activity of immune system against BC (23). In contrast, a robust correlation between reduced frequency of Treg cells and an unfavorable prognosis in post-surgical BC patients has been identified by Jóźwicki et al. (24). These opposite findings indicate that the specific role of Foxp3+ Treg cells in the development of BC remains to be fully elucidated.

We consulted the NCBI database and found that the rs3761548 polymorphism in the Foxp3 gene has been linked to malignancies such as colorectal cancer, gastric adenocarcinoma, and endometrial cancer (21). And the rs5902434 polymorphism has been proven to be associated with the onset of various diseases such as unexplained recurrent spontaneous abortion, allogenic hematopoietic stem cell transplantation, chronic obstructive pulmonary disease (25–27). Besides, both of the two loci are located in the promoter region of the Foxp3 gene, which might play a potential role in the regulation of Foxp3 expression. Consequently, the present study aimed to explore whether the two specific polymorphic variants (rs3761548 and rs5902434) contributed to the progression of BC within the Chinese Han population.

## 2 Materials and methods

### 2.1 Subjects and characteristics

This case-control study enrolled a total of 316 patients diagnosed with BC, with a mean age of 63.76 ± 12.14 years, as well as 643 healthy controls with a mean age of 44.64 ± 15.55 years, from the West China Hospital of Sichuan University between 2001 and 2012. The study protocol was approved by the hospital’s ethics committee, and all participants provided informed consent. Patients with a prior history of cancer, autoimmune or infectious diseases, or those who had undergone radiotherapy or chemotherapy were excluded, as these conditions could potentially confound the study results. Healthy controls were recruited from the hospital’s department of physical examination, and selected based on the absence of genetic relationships between participants. Individuals with a personal or family history of BC or other severe illnesses were also excluded from the control group. A follow-up protocol was established, consisting of telephone calls every six months for a duration of five years. The histopathological analysis was performed to confirm the presence of tumor tissues in resected specimens obtained from the patients. Clinical characteristic data were extracted from medical records and are detailed in **Table 1**.

**Table 1:** Characteristics of participated subjects.

Characteristics	Patients (n=316)	Controls (n=643)	P value
Gender			
Male	249 (78.8%)	275 (42.8%)	
Female	67 (21.2%)	368 (57.2%)	<0.001
**Age (years old, mean ± SD)**	63.76 ± 12.14	44.64 ± 15.55	<0.001
Smoking status			
Smoker	163 (51.6%)	–	
Non-smoker	153 (48.4%)	–	
Clinical stage			
I (Ta ~ T1N0M0)	156 (49.4%)	–	
II (T2N0M0)	84 (26.6%)	–	
III (T3N0M0, T4aN0M0)	34 (10.8%)	–	
IV (T4bN0M0, TnNnM0, TnNnMn, n ≥ 1)	20 (6.3%)	–	
NA	22 (6.9%)		
Tumor stage			
NMIBC	150 (47.5%)	–	
MIBC	166 (52.5%)	–	
Tumor grade			
High grade	182 (57.6%)	–	
Low grade	134 (42.4%)	–	

SD, standard deviation; NA, not available; n, corresponds to the number of individuals.

NMIBC, non-muscle-invasive bladder cancer; MIBC, muscle-invasive bladder cancer.

### 2.2 Gene selection and genotyping

Two SNPs (rs3761548 C/A, rs5902434 del/ATT) were identified from CHB population sample data of the HapMap Project through the SNPinfo software (28). Primers for polymerase chain reaction (PCR) were designed using Primer 3 web version 4.1.0. (http://primer3.ut.ee/) (29).

**Figure 1:**
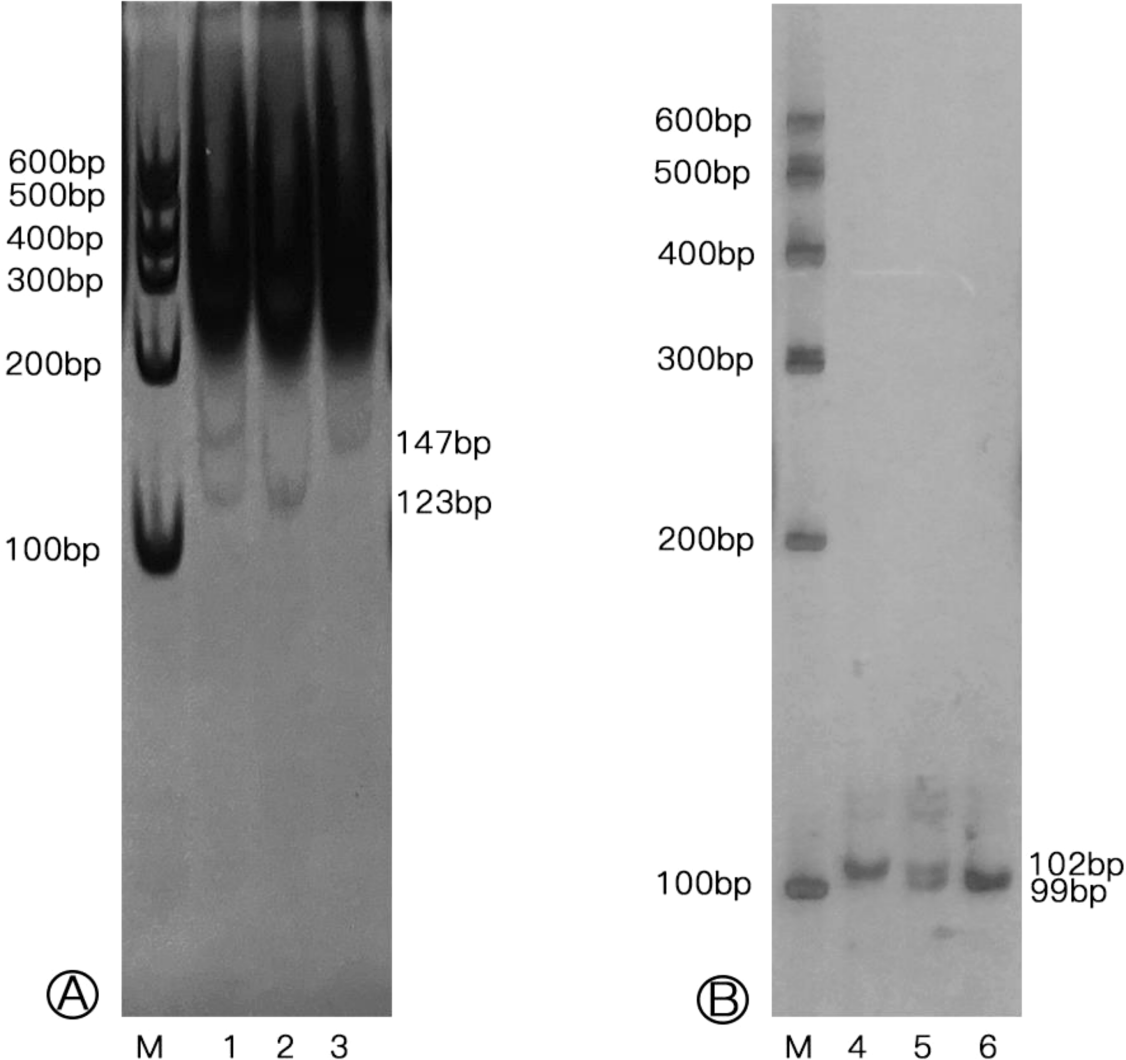
Genotypes of *Foxp3* rs3761548 and rs5902434 polymorphisms in individuals. **(A)** Genotypes of rs3761548 polymorphism (M: Marker; Lane 1: CA genotype; Lane 2: CC genotype; Lane 3: AA genotype); **(B)** Genotypes of rs5902434 polymorphism (M: Marker; Lane 4: ATT/ATT genotype; Lane 5: -/ATT genotype; Lane 6: -/- genotype).

Genomic DNA was extracted from a 200 μL sample of EDTA-anticoagulated peripheral blood taken from each participant, using a DNA isolation kit (BioTeke, China). The genotyping of the samples were performed using the polymerase chain reaction-restriction fragment length polymorphism (PCR-RFLP) technique. Amplification of genomic DNA fragments was conducted in a total volume of 10 μL, including 5 μL of 2x Power Taq PCR Master Mix (BioTeke, China), 4 picomoles of each primer, and 100 ng of genomic DNA. The specific primer sequences and the PCR conditions are detailed in **Table 2**. Subsequently, the PCR products for rs3761548 were digested using the restriction enzyme, as indicated in **Table 2**. In contrast, the rs5902434 fragments were directly separated by electrophoresis on a 15% polyacrylamide gel, and then the gels for both polymorphisms were stained using a 1.5 g/L silver nitrate solution (**Figure 1**). Finally, the genotypes were verified by DNA sequencing analysis. Approximately 10% of the samples were randomly chosen for repeat assays, which confirmed the initial results with 100% concordance.

**Table 2:** Primer sequences and reaction conditions for genotyping two SNPs.

SNPs	Primer Sequence	Wild/Mutated Allele	Annealing Temperature/°C	Restriction Enzyme	Product Size/bp
rs3761548	F:5′-GAAGGGCAAATTGAAGACCA-3′	C/A	60	PstI (37°C, 2h)	A (147)
	R:5′-GGTGCTGAGGGGTAAACTGA-3′				C (123 + 24)
rs5902434	F:5′-CCCTGCCCATGCATTAAGTA-3′	deletion/ATT	60	–	deletion (99)
	R:5′-TACCCAGCTACCGTGATTCC-3′				ATT (102)

SNP, single nucleotide polymorphism; bp, base pair; F, forward primer; R, reverse primer.

### 2.3 Statistical analysis

Data analysis was conducted using SPSS software, version 20.0 (SPSS Inc., Chicago, IL, USA). The online SNPstats software (www.snpstats.net/start.htm) was used to determine the disparities in allele and genotypic distributions between the case and control groups. It was achieved by directly counting genotype frequencies across various genetic models, including codominant, dominant, recessive, and over-dominant models (30). The Hardy-Weinberg equilibrium was assessed using a chi-squared test. Odds ratios (OR) and 95% confidence intervals (CI) were calculated to detect the correlation between specific genotypes and clinical characteristics. Univariate survival analysis was performed using Kaplan-Meier plots and the log-rank test, while multivariate survival analysis was conducted using the Cox proportional hazards regression model. P < 0.05 was set as the statistical significance threshold.

## 3 Results

### 3.1 Foxp3 genetic distribution and BC susceptibility

The allele and genotype distributions for the two SNPs adhered to the assumption of Hardy-Weinberg equilibrium (*P* > 0.05) and are presented in **Table 3** after adjusted by gender. In the case of rs3761548 C/A polymorphism, the frequency of A allele within BC patients was decreased compared to that in controls with statistical significance (18.5% versus 24.4%, P = 0.01, OR = 0.72, 95%CI = 0.56-0.93), and it was in line with the distribution of CA/AA genotypes in the dominant model (22.1% versus 34.8%, P = 0.003, OR = 0.61, 95%CI = 0.44-0.85), which indicated a significantly lower BC risk of CA/AA genotypes carriers. The heterozygous CA genotype of rs3761548 presented a distinctly decreased risk for BC in the codominant model (P = 0.001, OR = 0.42, 95%CI = 0.25-0.68) and the over-dominant model (P = 0.0003, OR = 0.43, 95%CI = 0.26-0.70) when in contrast with the homozygous CC/AA genotypes.

**Table 3:** The association between bladder cancer risk and the distribution of SNPs in *FOXP3* among patients and controls.

Model	rs3761548					rs5902434				
	Genotype	Controls	Patients	OR (95%CI) ^a^	*P* value ^a^	Genotype	Controls	Patients	OR (95%CI) ^a^	*P* value ^a^
		n (%)	n (%)				n (%)	n (%)		
Codominant	CC	419 (65.2%)	246 (77.8%)	1.00 (ref)		-/-	276 (42.9%)	162 (51.3%)	1.00 (ref)	
	CA	134 (20.8%)	23 (7.3%)	**0.42 (0.25-0.68)**	**0.001**	-/ATT	227 (35.3%)	59 (18.7%)	**0.61 (0.42-0.89)**	**0.01**
	AA	90 (14%)	47 (14.9%)	0.81 (0.54-1.21)	0.27	ATT/ATT	140 (21.8%)	95 (30.1%)	1.06 (0.75-1.49)	0.82
Dominant	CC	419 (65.2%)	246 (77.8%)	1.00 (ref)		-/-	276 (42.9%)	162 (51.3%)	1.00 (ref)	
	CA/AA	224 (34.8%)	70 (22.1%)	**0.61 (0.44-0.85)**	**0.003**	-/ATT+ATT/ATT	367 (57.1%)	154 (48.7%)	0.82 (0.62-1.10)	0.18
Recessive	CC/CA	553 (86%)	269 (85.1%)	1.00 (ref)		-/- + -/ATT	503 (78.2%)	221 (69.9%)	1.00 (ref)	
	AA	90 (14%)	47 (14.9%)	0.91 (0.60-1.36)	0.63	ATT/ATT	140 (21.8%)	95 (30.1%)	1.24 (0.90-1.71)	0.2
Over-dominant	CC/AA	509 (79.2%)	293 (92.7%)	1.00 (ref)		-/- + ATT/ATT	416 (64.7%)	257 (81.3%)	1.00 (ref)	
	CA	134 (20.8%)	23 (7.3%)	**0.43 (0.26-0.70)**	**0.0003**	-/ATT	227 (35.3%)	59 (18.7%)	**0.60 (0.42-0.85)**	**0.004**
Allele	C	972 (75.6%)	515 (81.5%)	1.00 (ref)		–	779 (60.6%)	383 (60.6%)	1.00 (ref)	
	A	314 (24.4%)	117 (18.5%)	**0.72 (0.56–0.93)**	**0.01**	ATT	507 (39.4%)	249 (39.4%)	1.02 (0.83–1.25)	0.89

OR, odds ratio; CI, confidence interval; n corresponds to the number of individuals. ^a^Adjusted by gender.

Bold faced values indicate a significant difference at the 5% level.

For the rs5902434 del/ATT polymorphism, a decreased susceptibility to BC was observed across the codominant and over-dominant models with statistical significance (codominant model: P = 0.01, OR = 0.61, 95%CI = 0.42-0.89; over-dominant model: P = 0.004, OR = 0.60, 95%CI = 0.42-0.85).

### 3.2 Foxp3 SNPs and subgroup analyses

Stratified analyses were conducted to examine the distribution of Foxp3 genotypes among BC patients relative to various demographic and clinical characteristic groups, including age (≤64 years and >64 years), gender (female and male), smoking status, tumor grade (high and low), as well as the presence of relapse and metastasis. The results are presented in **Table 4**. No significant differences were observed across most subgroups for the two SNPs, with the exception of gender and metastasis status. Regarding the rs3761548 polymorphism, male patients with the CA genotype demonstrated a higher risk of BC in contrast with females, with an adjusted OR of 16.16 (95%CI = 4.53-57.68) after adjusting for variables such as age, smoking status, tumor stage, grade, relapse, and metastasis. A similar pattern was observed for the del/ATT heterozygotes in the rs5902434 polymorphism, where the OR was 7.19 (95%CI = 3.31-15.61). Notably, considering the location of Foxp3 gene (located on X chromosome) and the shortage of female patient number, the results of gender analysis should be approached dialectically, because this might present a gender preference and lead a result with bias. Individuals homozygous for the ATT allele in rs5902434 (ATT/ATT) displayed a reduced risk of metastasis when compared to those with del/del and del/ATT genotypes, with an OR of 0.31 (95%CI = 0.13-0.74).

**Table 4:** Association between SNPs of *FOXP3* and patients’ clinical characteristics.

Characteristics	rs3761548			rs5902434		
	Dominant model (C/C vs. C/A+A/A)	Recessive model (C/C+C/A vs. A/A)	Over-dominant model (C/C+A/A vs. C/A)	Dominant model (-/- vs. -/ATT+ATT/ATT)	Recessive model (-/- + -/ATT vs. ATT/ATT)	Over-dominant model (-/- + ATT/ATT vs. -/ATT)
	OR (95% CI) ^a^	OR (95% CI) ^a^	OR (95% CI) ^a^	OR (95% CI) ^a^	OR (95% CI) ^a^	OR (95% CI) ^a^
Age (years old)						
≤64	1.00 (ref)	1.00 (ref)	1.00 (ref)	1.00 (ref)	1.00 (ref)	1.00 (ref)
>64	0.76 (0.42-1.38)	1.07 (0.53-2.16)	0.39 (0.14-1.09)	1.01 (0.61-1.66)	0.80 (0.46-1.37)	1.46 (0.73-2.92)
Gender						
Female	1.00 (ref)	1.00 (ref)	1.00 (ref)	1.00 (ref)	1.00 (ref)	1.00 (ref)
Male	2.06 (0.97-4.35)	**0.27 (0.08-0.99)**	**16.16 (4.53-57.68)**	**3.01 (1.52-5.97)**	0.51 (0.23-1.17)	**7.19 (3.31-15.61)**
Smoking status						
Non-smoking	1.00 (ref)	1.00 (ref)	1.00 (ref)	1.00 (ref)	1.00 (ref)	1.00 (ref)
Smoking	1.02 (0.54-1.95)	0.95 (0.46-1.94)	1.34 (0.36-5.01)	0.73 (0.43-1.24)	0.56 (0.31-1.00)	1.48 (0.68-3.20)
Grade						
High	1.00 (ref)	1.00 (ref)	1.00 (ref)	1.00 (ref)	1.00 (ref)	1.00 (ref)
Low	0.96 (0.46-2.02)	1.28 (0.53-3.09)	0.56 (0.16-1.92)	0.66 (0.36-1.21)	0.84 (0.44-1.62)	0.64 (0.28-1.43)
Relapse						
No	1.00 (ref)	1.00 (ref)	1.00 (ref)	1.00 (ref)	1.00 (ref)	1.00 (ref)
Yes	1.71 (0.86-3.38)	1.25 (0.58-2.66)	3.46 (0.88-13.65)	1.20 (0.70-2.05)	1.01 (0.56-1.80)	1.41 (0.65-3.03)
Metastasis						
No	1.00 (ref)	1.00 (ref)	1.00 (ref)	1.00 (ref)	1.00 (ref)	1.00 (ref)
Yes	0.64 (0.26-1.59)	0.49 (0.17-1.42)	1.20 (0.25-5.84)	0.50 (0.22-1.11)	**0.31 (0.13-0.74)**	1.89 (0.57-6.23)

OR, odds ratio; CI, confidence interval. ^a^Adjusted by age, gender, smoking status, stage, grade, relapse and metastasis.

Bold faced values indicate a significant difference at the 5% level.

### 3.3 Foxp3 genotypes and survival analyses

In the current study, a total of 316 BC patients were enrolled in a follow-up plan that included telephone calls every 6 months for a period of 5 years. By the end of the follow-up period, 51 patients (16.1%, NMIBC: 13; MIBC: 38) were dead. To assess overall survival (OS), Kaplan-Meier analysis and Cox regression analysis was conducted, revealing no significant correlation between the OS rate of BC patients and the two SNPs (P > 0.05).

It is well recognized that the prognosis of NMIBC and MIBC is significantly different following treatment. To delve deeper into the outcomes of BC patients, this study conducted a Cox multivariate survival analysis to investigate the outcomes of NMIBC and MIBC patients. The findings are presented in **Table 5**. According to the Cox multivariate survival analysis, individuals who were homozygous for the AA genotype at rs3761548 demonstrated a markedly lower OS rate compared to those with the CC/CA genotypes (P = 0.03, HR = 5.89, 95%CI = 1.23-28.15), after adjusting for factors such as age, gender, smoking status, tumor grade, metastasis, and clinical stage. No significant association was observed with the rs5902434 polymorphism.

**Table 5:** Association between SNPs in *FOXP3* and survival for NMIBC and MIBC patients.

SNP/genotype	NMIBC				MIBC			
		Alive/Dead	HR (95%CI) ^a^	*P* value ^a^	Alive/Dead	HR (95%CI) ^a^	*P* value ^a^
rs3761548								
	CC	119/10			90/27		
	CA	12/0			7/4		
	AA	22/3			15/7		
	Dominant		3.23 (0.70 – 14.87)	0.13		1.15 (0.53 – 2.51)	0.72
	Recessive		**5.89 (1.23 – 28.15)**	**0.03**		1.28 (0.52 – 3.18)	0.59
	Over-dominant		**-**	–		0.92 (0.27 – 3.12)	0.89
rs5902434								
	-/-	71/7			68/16		
	-/ATT	37/0			15/7		
	ATT/ATT	45/6			29/15		
	Dominant		1.07 (0.29 – 3.93)	0.92		0.99 (0.45 – 2.22)	0.99
	Recessive		2.09 (0.63 – 6.89)	0.23		0.99 (0.45 – 2.07)	0.99
	Over-dominant		–	–		1.00 (0.28 – 3.54)	0.99

HR, hazard ratio; CI, confidence interval. ^a^Adjusted by age, gender, smoking status, tumor grade, metastasis and clinical stage.

Bold faced values indicate a significant difference at the 5% level.

## 4 Discussion

Foxp3 is considered a hallmark molecule of Tregs, functioning as a transcriptional regulator that controls the activity of Treg by directly regulating the expression of multiple genes (19). Mutations in the Foxp3 gene could potentially lead to a decrease in Treg cell function as well as the secretion of inhibitory cytokines, leading to a disruption in the immune homeostasis and the development of serious autoimmune diseases (20). In recent years, there has been a growing focus on the regulation and influence of Foxp3 on disease pathophysiology. Genetic polymorphisms with the Foxp3 gene may functionally or quantitatively alter the protein, thereby influencing the risk of developing certain disease.

Previous studies have explored the interaction between cancer risk and the polymorphism of Foxp3 gene. In 2013, He et al. demonstrated that the A allele of Foxp3 rs3761548 increased the risk of non-small cell lung cancer (31). Subsequently, in a study by Chen et al., the Foxp3 gene polymorphism at rs3761548 was found to be a contributing factor to the high susceptibility to colorectal cancer within the Chinese population (32). In 2019, Nazanin et al. also found that the AA genotype and A allele of Foxp3 rs3761548 were linked to a higher risk of prostate cancer incidence (33).

In contrast, a study on endometrial cancer has reported a positive effect of Foxp3 polymorphisms, where the CA heterozygotes of rs3761548 were found to have a protective role, and the ATT/ATT genotype of rs5902434 was associated with a reduced risk of endometrial cancer (34). The findings above suggest that the influence of Foxp3 polymorphisms on cancer susceptibility may vary significantly between different types of cancer. To the best of our knowledge, the current study is the first investigation into the association between Foxp3 gene polymorphisms and both the susceptibility to and prognosis of BC. Our results revealed that heterozygotes for both rs3761548 and rs5902434 polymorphisms are linked to a reduced risk of BC susceptibility, which was in line with the aforementioned study even the gender preference existed (males occupying 80% of the incidence). Additionally, we observed that AA homozygotes for the rs3761548 polymorphism had a notably lower OS rate among BC patients which was coincidence with the results of non-small cell lung cancer, colorectal cancer, and prostate cancer. The paradoxical prognostic effects of Foxp3 gene polymorphisms on tumors were in line with the role of Tregs on BC.

Similar self-contradictory phenomenon has been observed in research concerning the relationship between Foxp3 expression and BC prognosis. Horn et al. pointed out that an increased ratio of Foxp3/CD3 was associated with slightly shorter OS in BC patients (35). In contrast, Winerdal et al. found that while Foxp3 expression in BC cells correlated with decreased long-term survival, a higher infiltration of Foxp3 +  tumor-infiltrating lymphocytes (TILs) was correlated with better survival outcomes, highlighting the complex role of TILs in cancer progression (36). Furthermore, in 2018, the same researchers suggested that Treg-mediated suppression of matrix metalloproteinase 2 within the MIBC tumor microenvironment may be a potential mechanism underlying the positive prognostic impact of tumor-infiltrating Tregs (22).

Within the tumor microenvironment, Tregs are known to enhance the proliferation and activation of immune cells, which can lead to improved patient survival rates. It suggests that cellular interactions within the tumor microenvironment may exert a greater impact on patient survival than the intrinsic subtypes of the tumor itself (37). In the present study, we demonstrated that mutations in the Foxp3 gene were associated with a poorer prognosis in NMIBC, which might be attributable to the potential dysfunction of Tregs resulting from the disrupted expression of the mutated Foxp3 gene.

In conclusion, this study presents an initial exploration into the relationship between Foxp3 polymorphisms and BC. Our findings indicate that Foxp3 polymorphisms may be associated with the risk of BC susceptibility, and that rs3761548 could potentially serve as an independent risk factor for the OS rate. However, this study has several limitations. At first, the sample size, especially the female patients, was insufficient because of the higher BC incident rate of male than that of female and the genetic patten of X-linked gene, which might affect the reliability and veracity of the results, although we have conducted Cochran’s and Mantel-Haenszel statistics during analyses to adjust the gender parameter. Secondly, the lack of information on the smoking status of controls might introduce bias in this study since smoking is a known risk factor for BC. And more, the definitive impact of these two SNPs on the protein level of Foxp3 remains unclear. Therefore, further research is necessary to comprehensively elucidate this relationship.

## Data Availability

The raw data supporting the conclusions of this article will be made available by the authors, without undue reservation.
